# Strengthening the regulatory system through the implementation and use of a quality management system

**DOI:** 10.26633/RPSP.2017.12

**Published:** 2017-02-08

**Authors:** Reinhold Eisner, Rakeshkumar Patel

**Affiliations:** 1 Biologics and Genetic Therapies Directorate Health Canada OttawaOntario Canada

**Keywords:** Quality control, quality improvement, Canada, Control de calidad, mejoramiento de la calidad, Canada

## Abstract

Quality management systems (QMS), based on ISO 9001 requirements, are applicable to government service organizations such as Health Canada’s Biologics and Genetic Therapies Directorate (BGTD). This communication presents the process that the BGTD followed since the early 2000s to implement a quality management system and describes how the regulatory system was improved as a result of this project. BGTD undertook the implementation of a quality management system based on ISO 9001 and containing aspects of ISO 17025 with the goal of strengthening the regulatory system through improvements in the people, processes, and services of the organization. We discuss the strategy used by BGTD to implement the QMS and the benefits that were realized from the various stages of implementation. The eight quality principals upon which the QMS standards of the ISO 9000 series are based were used by senior management as a framework to guide QMS implementation.

## RESUMEN

De acuerdo con las normas ISO 9001, los sistemas de gestión de la calidad (SGC) son aplicables a las entidades de la administración pública, como la Dirección de Insumos Biológicos y Terapias Genéticas de Salud Canadá (BGTD por su sigla en inglés). En esta comunicación se describe el proceso que siguió la BGTD desde principios del siglo XXI para instituir un sistema de gestión de la calidad y las mejoras incorporadas como resultado de este proyecto. La BGTD se propuso instaurar un sistema de gestión de la calidad conforme a la norma ISO 9001 y algunos aspectos de la ISO 17025 con el propósito de fortalecer el sistema de reglamentación mejorando los recursos humanos, los procesos y los servicios de la entidad. En esta comunicación, describimos la estrategia empleada por la BGTD para aplicar el SGC y los beneficios logrados en las diversas etapas de su aplicación. A modo de guía en la aplicación del SGC, el personal directivo superior utilizó como marco los ocho principios de gestión de la calidad en los que se basa la serie de normas ISO 9000.

In this communication we wish to present the process that the Biologics and Genetic Therapies Directorate in Canada followed to implement a quality management system (QMS) and describe how the regulatory system was improved as a result of the benefits achieved during this project.

The life cycle of a drug in Canada, from submission for clinical trials until it is withdrawn from the market, is regulated by Canada’s regulatory authority, Health Canada. Health Canada’s role is to ensure that the drug complies with the regulations and laws enacted by the Government of Canada and to ensure that the people of Canada have timely access to safe, effective, and high-quality drugs.

Health Canada’s Biologics and Genetic Therapies Directorate (BGTD), which has specific responsibility for the regulation of biologic drugs and radiopharmaceuticals, reflects this role in its quality policy: “to carry out work in a predictable, transparent, and open manner with a commitment to the continual improvement of the people, processes, and services of the organization, and in a manner that provides timely risk-based decisions, and enabling access to safe, effective, and quality Biologics and Radiopharmaceuticals for the people of Canada.”

In the early 2000s, BGTD conducted a review of its activities related to testing laboratories and submissions review. The review concluded that an appropriate organizational structure and processes to conduct its activities were present and functioning. It also identified areas that could benefit from improvement. Suggested improvements included the following: encouraging the identification and solution of problems, potentially reducing operating costs, creating awareness of the need for training, encouraging a focus on internal processes, enhancing the reputation of the organization, improving the effectiveness and efficiency of internal operations, enhancing client/customer satisfaction and communication, potentially reducing re-analysis of samples, improving validity and trustworthiness of test data, and clarifying the definition of roles, responsibilities, and authorities. The BGTD Management Committee (DMC) made the strategic decision to move forward with a formal quality management system across BGTD. The QMS chosen was based on ISO 9001:2008 (1), with integration of the requirements of ISO/IEC 17025:2005 – General Requirements for the Competence of Testing and Calibration Laboratories (2). The latter is applicable to the BGTD laboratories and covers technical competence requirements that are not covered by ISO 9001.

## BGTD’S STRATEGY FOR IMPLEMENTING A QMS

The quality management system standards of the ISO 9000 series are based on eight quality management principles (3). These principles were used by senior management as a framework to guide the BGTD in implementing a QMS and to work toward the desired performance improvement.

The first phase in implementation of a QMS was to recognize the importance of the *leadership* principle. This principle states: “Leaders establish unity of purpose and direction of the organization. Leaders should create and maintain the internal environment in which people can become fully involved in achieving the organization’s objectives.” The BGTD ensured the leadership aspect would be present and active by making a commitment that the DMC and management be fully involved and supportive in all the stages of the implementation.

**FIGURE 1:**
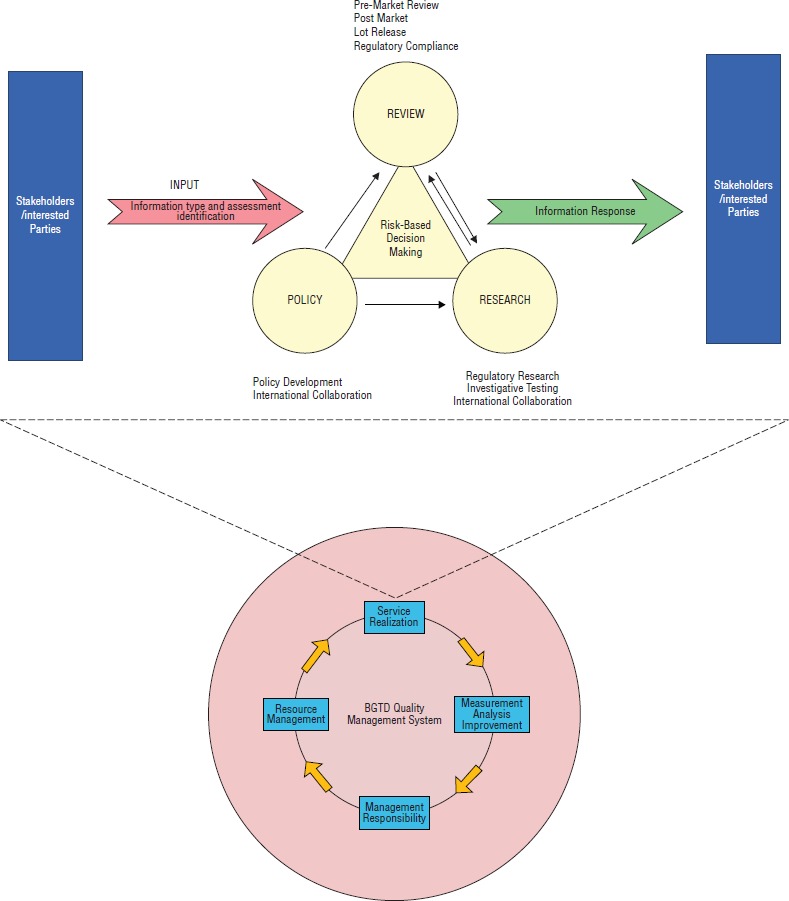
Working model of the quality management system implemented by Health Canada’s Biologics and Genetic Therapies Directorate (BGTD)

The second phase was application of the *system approach for management* principle, which states: “Identifying, understanding, and managing interrelated processes as a system contributes to the organization’s effectiveness and efficiency in achieving its objectives.” Typically, the system approach to management looks at an organization as a complex entity. It is therefore important to identify, understand, and manage individual processes and their interactions within the organizational workflow. BGTD developed a QMS model consisting of four main activities: Service Realization, Measurement Analysis and Improvement, Management Responsibility, and Resource Management (see Figure 1). The service realization activity of BGTD is a risk-based decision making activity comprising three interacting components—review, policy, and research—that encompass the core business processes.

The third phase was application of the *process approach* principle. It states: “A desired result is achieved more efficiently when activities and related resources are managed as a process.” The process approach required that individual processes be defined, their inputs and outputs determined, process owners and customers identified, and the interfaces with the organization’s function identified. BGTD identified eight processes as being essential to the business (see Figure 1).

Once the activities in the process approach phase were well under way, the next phase, *continual improvement,* was gradually introduced. This long-term objective of continual improvement of performance was instituted through the use of management review meetings, internal audits, identification of nonconformities, and application of the continual improvement requirements identified in ISO 9001 section 8.5 (1).

The other principles—involvement of people, factual decision making, and customer focus—co-evolved with the principles of leadership, system approach to management, process approach, and continual improvement as they were used in establishing a functioning QMS.

## BENEFITS REALIZED DURING IMPLEMENTATION OF A QMS

### Leadership approach

The senior management of BGTD, the DMC, recognized the value of the leadership principle and created an environment of uniform understanding of the organization’s direction by establishing the quality policy and quality objectives and communicating them to all the employees of BGTD. Continued management commitment to improving the system was demonstrated by participation in the implementation and maintenance activities of the QMS, such as approving the procedures and processes of BGTD, participating in quality audits and management review activities, supporting implementation of the quality objectives, and identifying and allocating the necessary resources to achieve these goals.

These leadership activities resulted in the creation of a common approach to quality and a staff that, through improved understanding of the goal of the organization, was more motivated, competent, and stable. A side benefit to this approach was the evolution of improved communications between the various levels of BGTD.

### System approach to management

While processes were in place when the BGTD started this initiative, the overall picture of how the processes interacted was not fully understood, which resulted in various divisions functioning more or less independently and with lowered effectiveness and efficiency in delivering the services of BGTD. Development of the business system model (Figure 1) provided senior management with a tool to gain a clear, detailed understanding of the business processes, the interdependencies of the system processes, the operation of specific activities within the system, and the necessary roles and responsibilities. With this improved understanding, BGTD management was able to focus their efforts on integrating and aligning the core business processes. The critical organizational capabilities and resource constraints were more readily identified, as were sources of error and areas of potential improvement. Management was therefore able to allocate resources based on factual need rather than perceived need, which ultimately resulted in improved effectiveness in service provision and a reduction in operating costs.

### Process approach

Understanding of the operation of business-essential processes was achieved by developing process maps and identifying the main activities within the process, the inputs and outputs of each activity, and the documentation required or generated. This undertaking was accomplished with the help of employees directly involved in the corresponding activities. During this phase, the ISO 9001 requirements for document and record control were being addressed through the development of operating procedures for consistent service delivery, and software systems were implemented to ensure easy access to information. Examples were QSi for life-cycle management of procedures, instructions, and templates, and LIMS for maintenance of information and approval test samples and results.

The process approach constituted the bulk of the BGTD’s effort in this initiative. From the process maps, BGTD identified a number of issues that needed further clarification or improvement, such as the following: a) interactions between divisions within the process and the consequences of a breakdown in these interactions, b) clarification of roles and responsibilities for each activity and position to avoid overlap or duplication of work, c) increased consistency in how work was done, d) identification of records that needed to be generated and retained (forms, templates, supporting documentation), and e) the need for better training of employees in their activities.

Addressing these issues benefited management, employees, and BGTD as a whole. The benefits realized were:

Management: a) the ability to focus on and prioritize improvement opportunities; b) improved capability to allocate the appropriate resources for each activity; c) the ability to respond quickly to changes in work demands by shifting resources while minimizing risks to other areas.

Employees: a) better understanding of their roles and responsibilities, how they interact with others, and the overall impact of their activities on the process; b) improved internal communication between employees and departments; c) consistency in performing their work through the use of standard operating procedures; d) awareness that the procedures are dynamic and that changes may be initiated by anyone; e) increased confidence by the laboratories in their results owing to a clearer understanding of the accuracy and precision of the testing.

BGTD: a) identification of appropriate performance indicators to support cost recovery; b) improved capability to analyze the strengths and weaknesses of the regulatory review process; c) improved ability to identify essential documentation for business purposes (standard operating procedures, forms, and policies); and d) improved identification of process weaknesses and improvement measures using the continual improvement tools and auditing of the processes.

### Continual improvement

Prior to the QMS, improvements to BGTD’s operations and service delivery were limited to the various work units, with little consideration of how the changes would affect other areas of the organization. The QMS provided staff with a suite of tools for continual improvement, such as document change requests, nonconformance reports, corrective/ preventive actions, and change control, whereas management had tools such as management review and internal/ external audits. These tools provide the means for staff members to express their ideas, as well as opportunities to improve and to identify nonconformities, while providing management the means to determine overall impacts on the effectiveness/efficiency of the process and system.

The BGTD benefited from application of the continual improvement activities and analysis of the resulting information in several ways. It was able to: a) identify areas requiring improvement, b) set realistic, measurable goals for improvement by means of the management review process and factual analysis of data and information, c) monitor performance of the processes with a view to improving the work, and d) standardize employee training through identifying the basic requirements for each position, determining the training needs for each employee, creating a training program, and evaluating the effectiveness of the training.

### Involvement of staff

Prior to implementing the QMS, BGTD employees were aware of activities directly related to their work but were less aware of how their activities impacted on the rest of the process. This situation resulted in a general dissatisfaction in the way things were being done, frustration over not being able to change or improve things, and lack of a clear understanding of management’s expectations. During implementation of the QMS, employees received training designed to improve performance in their regular activities, which increased awareness of their role in the overall processes and increased participation in process improvement.

By being fully involved in implementation of the QMS, employees learned that they had the ability to initiate change and improve processes. This resulted in a culture change; employees began to look forward to promoting their ideas on how to improve the process rather than expressing discontent and feeling powerless to effect changes. Other benefits realized by the employees were: a) increased clarity about their roles and responsibilities within the process and system, b) more satisfaction with their work, c) more motivation, commitment, and involvement within the organization, d) better understanding of the importance of their contribution and role in the organization, e) increased ownership of problems and their responsibility for solving them, f) active pursuit of opportunities to enhance their competence, knowledge, and experience, and g) more willingness to discuss problems and issues openly.

### Customer focus

As a regulatory agency, the BGTD does not have customers in the usual business sense. Management recognized that they needed to be responsive to their stakeholders and, accordingly, obtained feedback from the stakeholders by means of regular bilateral meetings, regulatory meetings, pre-submission meeting requests, and on-request meetings.

With the implementation of the QMS, BGTD increased emphasis on customer feedback by identifying the forms of feedback present in the organization and making efforts to monitor and analyze these feedbacks. Feedback results were communicated to the employees to make them aware of the expectations of stakeholders. These changes benefited the BGTD by enabling it to: a) respond more quickly and flexibly to changes in regulatory requirements, b) make more effective use of the organization’s resources to enhance stakeholder satisfaction, c) link stakeholder needs and expectations to the objectives of the organization, and d) have stakeholder needs and expectations communicated and understood throughout the organization.

### Factual approach to decision making

Initially, development of organizational strategies was based mostly on observed requirements for resources and material and partially understood process relationships. With the QMS requirements that processes be monitored and evaluated, BGTD instituted mechanisms to gather and analyze data for critical processes on a regular basis. Yearly reports on these data are presented to the DMC during the management review meeting, providing the DMC a full picture of the organization’s status and helping it to make fact-based decisions on future directions. Utilizing this factually based approach helps BGTD management to: a) make wellinformed decisions and develop strategies based on factual analysis, balanced with experience and intuition; b) evaluate the effectiveness of past decisions through factual records and trend analysis; c) review, challenge, or change opinions and decisions; and d) make the appropriate data accessible to those who need it (for planning, resource allocation, budgeting, etc.).

## CONCLUSION

The implementation and use of a QMS within BGTD resulted in a regulatory system that is more robust, responsive, and adaptable to change. The robustness improved through increased opportunities for employees and stakeholders to identify areas and processes requiring improvement, factual analysis of the opportunities, and use of continual improvement processes. By having a welltrained, stable, and involved staff and a management aware of process interactions, the ability to respond to changing work demands was greatly improved. Better knowledge of stakeholders’ needs, use of continual improvement tools, management review of the system, and internal/external audits provide the means to identify and implement changes to the system. Certification to ISO 9001 and ISO/IEC 17025 has contributed significantly to BGTD’s international reputation as a reliable regulatory body with high consistency of service delivery.
